# Association of sarcopenia and physical activity with femur bone mineral density in elderly women

**DOI:** 10.20463/jenb.2016.03.20.1.8

**Published:** 2016-03-31

**Authors:** Inhwan Lee, Changduk Ha, Hyunsik Kang

**Affiliations:** 1 College of Sport Science, Sungkyunkwan University, SuwonKorea

**Keywords:** femur bone mineral density, sarcopenia, physical activity, elderly women

## Abstract

**[Purpose]:**

This study examined the association of femur bone mineral density (BMD) with body composition and physical activity in elderly women.

**[Methods]:**

This was a cross sectional study involving 119 women with mean age of 73.1±5.5 years. Body composition parameters including body mass index (BMI), percent of body fat (%BF), appendicular skeletal muscle mass (ASM) index and femur BMD was measured by dual-energy X-ray absorptiometry (DXA). Physical activity was assessed by the uniaxial accelerometer for 7 consecutive days including weekends. Based on femur BMD T-scores, subjects were classified as optimal group, osteopenia group, and osteoporosis group. Based on ASM index, subjects were classified as normal group and sarcopenia group. According to WHO recommendations of physical activity for elderly, the subjects were classified as active group or inactive group. Logistic regression analyses were used to determine the odds ratio (OR) for osteopenia and osteoporosis.

**[Results]:**

There were linear decreases for body composition parameters including weight (*P*=.023), BMI (*P*=.039), lean mass (*P*=.032), ASM index (*P*=.007) and physical activity parameters including daily of step (*P*<.001), low intensity physical activity (*P*<.001), moderate intensity physical activity (*P*=.001) across femur BMD levels. Compared to the normal group (OR=1), the sarcopenia group had a significantly higher OR (OR=4.823; *P*=.042), and the inactive group had a significantly higher OR (OR=5.478; *P*=.005) having osteopenia and osteoporosis when compared to the active group (OR=1).

**[Conclusion]:**

The findings of this study suggested that physical activity along with a healthy nutrition should be promoted as a preventive strategy against osteopenia and osteoporosis in elderly women.

## INTRODUCTION

Aging population is globally progressing due to the decline in birth rate and extension of the average life span. In Korea, 65 years of age or older of the total population reached 12.7% based on 2014 and is expected to increase by 27.6% in 2034 at the current rate^1^. This aging population phenomenon induces a rapid increase in the prevalence of age-related diseases, among which degenerative musculoskeletal diseases including osteoporosis has been recognized as a serious problem over the years. Recent data indicates that osteoporosis treated cases continued to increase in the population aged over 50sover the past five years and has reportedly increased by about 75% in the population over age 70, as compared to five years ago. Also, according to the ratio of male and female osteoporosis treated people, the ratio of females over 50s is more than 10 times higher than that of male, so it seems that a social concern for postmenopausal women and the elderly is required^2^.

Osteoporosis refers to the likelihood of fractures due to the weakened strength of the bones and is defined as a skeletal disorder characterized by an abnormality of micro-structure and a reduction of bone mass3. Osteoporosis is often known as “the silent thief’ because fracture or secondary structural changes occur without symptoms. Thus, people with osteoporosis easily have a high risk of fracture even from a small external force4. Normal people can maintain bone density because bone resorption that is a removal process of new bone tissue and osteogenesis that is a generation process of the new bone tissue occur in balance. However, osteopenia could occur because bone turnover does not occur smoothly with age. In particular, estrogen deficiency of the postmenopausal women results in an imbalance of the bone resorption and bone formation promoting factors with changes in serum calcium concentration. This can lead to rapid changes in bone metabolism resulting in osteoporosis^5^. In addition, several recent studies reported that body composition, weight, alcohol consumption, amount of sunshine, nutritive condition, eating habits, and physical activities etc. have a significant effect on the bone mineral density (BMD), and the modifiable lifestyle factors were reported as a main cause of the osteoporosis^6,7^. Among them, changes in the body weight reportedly play an important role in bone metabolism. Precise biological mechanism of the body weight and bone metabolism remain unknown; however, weight loss inhibits the regeneration of the bone by reducing the mechanical loading passed to the bone and induces osteopenia by causing hormonal changes^8,9^.

In a previous study, Fu et al. reported different associations of fat mass and fat distribution with BMD in pre- and postmenopausal Chinese women aged 18 to 79 years^10^. The results of the survey on the association between the body composition and the BMD of each body part in 210 Asian menopausal women conducted by Ho-Pham et al. have reported that there is a significantly positive correlation between body weight, body mass index (BMI), muscle quantity, and the amount of body fat and the BMD of each body part^11^. The results of the survey on the correlation between the body composition and spinal fracture in the domestic 907 menopausal women conducted by Kim et al. have reported that body weight and BMI have a significantly positive correlation with lumbar BMD, but waist circumference and percent of body fat have a negative correlation with lumbar BMD^12^. Collectively, the studies suggest that the BMD in postmenopausal women is likely to have a significant association with body composition and changes in body weight, but this result is limited to only postmenopausal women. Thus, the studies on the elderly women over the 10 years after the onset of the menopause have not been conducted yet. Thus, additional studies on the role of lean fat that is a subordinate concept of the body weight and body fat play on changes in the BMD is necessary, since these can be a risk for obesity and cardiovascular diseases. In addition, physical activities have been in the spotlight as an effective method that can prevent and delay osteopenia in elderly women at an early age regardless of its form^13^. The development of modern society has resulted in a phenomenon of reduced time for physical activities and increased sedentary activities, which accelerates the onset of the degenerative diseases caused by normal aging. Hence, the importance of the regular physical activities is emphasized for attaining a healthy and stable quality of life physically and mentally^14^. In addition, regular physical activities in an old age reportedly play a positive role in preventing and delaying osteoporosis by inducing outdoor activities, increasing the synthesis of vitamin D and controlling the biochemical markers of bone metabolism such as osteocalcin^15^. Previously, the results of the 15-year follow-up study on the association between physical activities time and the osteopenia of each body part in 8,560 Europe menopausal women conducted by Rikkonen et al. indicated that progress of femur osteopenia is much slower in the group who spent time for the weekly physical activities and more than 90 minute physical activities can be an effective way to maintain BMD in menopausal women^16^. Also, Muraki et al. in the survey on the association between lifestyle factors and the lumbar BMD in 632 Asia elderly women reported that physical activity levels have a significantly positive correlation with lumbar spine BMD. Kim et al. conducted a study on correlation between leisure physical activities and lumbar and femoral BMD in 2,267 domestic menopausal women; they reported that the time and intensity of the leisure physical activities have a significantly positive correlation with the lumbar and femoral BMD^17,18^. According to these previous domestic and foreign studies, BMD in menopausal women is related to physical activities, but physical activity survey method in most previous studies is limited to the questionnaire alone. Thus, the role of the physical activities need verification by quantification of physical activity levels in the osteopenia elderly women with physical changes caused by menopause.

These results collectively suggest that BMD in elderly women is associated with the body composition and physical activities, but further studies on the elderly women with sufficient physical changes after menopause is required because most of the previous studies are limited to menopausal women. In addition, the relationship between lean fat that is a subordinate concepts of the body composition and body fat with BMD needs to be determined, in order to present the role and recommended levels of the physical activities for the osteopenia in the elderly women because most of the surveys on physical activities are limited to the questionnaire alone.

## METHODS

### Study subjects

The 131 elderly women aged 65 years and over with no special medical diseases and able to perform normal daily living activities were selected as study subjects. After all experiments were terminated, 119 people except for 12 people with missing data were selected as final subjects. Also, the subjects were involved in the experiment after receiving a written informed consent form (ICF) after detailed written and oral explanation about the purpose and progress methods of the study. The characteristics of the subjects were presented in Table 1. This study was conducted under the approval (SKKU-IRB-2014-01-002) of the S University Institutional Review Board (IRB).

**Table 1.** Physical characteristics of the participants (M±SD)

Variables	All (n=119)
Femur BMD (g/cm^2^)	0.8128 ± 0.1111
Age (yrs)	73.1 ± 5.5
Height (cm)	152.1 ± 5.0
Weight (kg)	56.8 ± 8.0
BMI (kg/m^2^)	24.9 ± 3.4
Body fat (%)	36.1 ± 6.3
Lean mass (kg)	34.7 ± 3.3
WC (cm)	84.0 ± 8.6
ASM index (kg/m^2^)	5.9 ± 0.6
Postmenopause (yrs)	49.5 ± 4.4

BMD: bone mineral density; BMI: body mass index; WC: waist circumference; ASM: appendicular skeletal muscle mass

### Study Method

#### 1) Body composition and BMD measurement

Automatic meter (DS-102, Jenix, Korea) was used for the height. The overall body composition such as body weight, body fat percentage, and lean fat percentage etc. was measured by using Hv-ps7681 (GE medical systems Lunar, USA) with Dual-energy X-ray Absorptiometry (DXA) principle in the right supine position after changing into comfortable clothes without metal materials. BMD in Femur total was measured in g/cm^2^ unit. Also, BMI was calculated using the weight / height (kg/m^2^). Investigators who had received trainings measured waist circumference at the midpoint of the lower border of rib cage and top of iliac crest by using anthropometric tape measure. Also, appendicular skeletal muscle mass index (ASM index) was calculated using the limb muscle amount / height (kg/m^2^) formula.

#### 2) Physical activities

In this study, all subjects were asked to wear Kenz Life corder accelerometer (Suzuken Co, Ltd, Nagoya, Japan) in the right waist site for 9 days except for the shower time in order to measure an individual’s physical activity. The data of a total of 7 days except for the date of wearing the device and removing it was analyzed. Accelerometer (Suzuken Co, Ltd, Nagoya, Japan) used in this study allows to download the PC data with uniaxial accelerometer and is printed and viewed as a result sheet by connecting exercise intensity, the number of walking, calorie consumption, and physical activities over time etc.. These are equivalent to the MET concept per four seconds to the body fat analyzer and have the advantage that subjects naturally participate in the measurement because of a small and light characteristics.

### Data processing methods

All data in this study was expressed as the mean and standard deviation (mean ± SD). The trend with or without linear trend between the measured variables was verified by using the contrast polynomial of the one-way analysis of variance after subdividing into the normal group (T-score > -1.0), osteopenia group (-1.0 ≥ T-score > -2.5), and osteoporosis group (T-score ≤ -2.5) based on the T score of the femur density^3^. A relatively odds ratio (OR) of the osteopenia and osteoporosis was calculated according to the loss of muscle mass after classifying into the normal group with more than 5.27kg/m^2^ and the muscle loss group with less than 5.27kg/m^2^ through the ASM index^4^. A relative risk was calculated by dividing into the active group that meet the elderly physical activities (moderate or vigorous physical activities for 150 minutes) recommended by WHO and inactive group that does not. SPSS-PC (version 18.0) was used for all statistical analysis and the significance level for the hypothesis verification was set at α = .05.

## RESULTS

### Comparison of the body composition and obesity indicators according to the BMD levels

**Figure 1.**
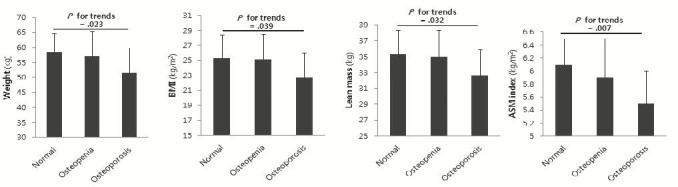
Comparison of body composition parameters according to normal, osteopenia and osteoporosis groups

Table 2 showed the comparison results of the body composition and obesity indicators among groups classified according to the level of the BMD. The result indicated a significant linear trend among groups in age (*P*<.001), weight (*P*=.023), BMI (*P*=.039), lean mass (*P*=.032), and ASM index (*P*=.007) and no significant differences among groups in the remaining variables (Figure 1).

**Table 2.** Comparison of body composition variables between normal, osteopenia and osteoporosis (M±SD)

Variables	Normal (n=35)	Osteopenia (n=70)	Osteoporosis (n=14)	*P* for linear trends
Femur BMD (g/cm2)	0.9367±0.0760	0.7825±0.0481	0.6546±0.1073	<.001
Age (yrs)	71.7±5.3	72.4±4.5	80.3±6.0	<.001
Height (cm)	152.3±4.7	152.1±5.2	151.6±4.4	.898
Weight (kg)	58.3±6.5	57.1±8.3	51.5±8.5	.023
BMI (kg/m2)	25.3±3.1	25.1±3.4	22.7±3.3	.039
Body fat (%)	36.7±6.2	36.3±6.1	33.6±7.7	.264
Lean mass (kg)	35.3±3.0	34.9±3.4	32.6±3.3	.032
WC (cm)	83.6±9.3	84.3±8.3	83.8±8.8	.934
ASM index (kg/m2)	6.1±0.4	5.9±0.6	5.5±0.5	.007
Postmenopause (yrs)	50.7±3.9	49.0±4.8	49.0±3.0	.180

BMD: bone mineral density; BMI: body mass index; WC: waist circumference; ASM: appendicular skeletal muscle mass

### The comparison of physical activity parameters according to the BMD levels

**Figure 2.**
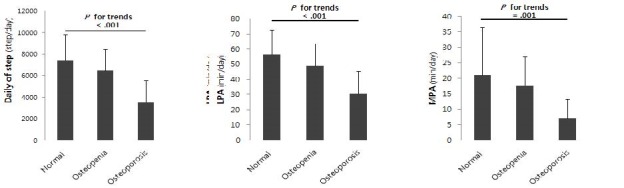
Comparison of physical activity parameters according to normal, osteopenia and osteoporosis groups

Table 3 showed the comparison among groups subdivided by the levels of the BMD. The result indicated a linear trend in the daily of step (*P*<.001), low intensity physical activity (*P*<.001), and moderate intensity physical activity (*P*=.001) and no significant difference among groups in high intensity physical activity (Figure 2).

**Table 3.** Comparison of physical activity parameters between normal, osteopenia and osteoporosis (M±SD)

Variables	Normal (n=35)	Osteopenia (n=70)	Osteoporosis (n=14)	*P* for linear trends
Daily of Step (step/day)	7411.8±2386.6	6499.3±1963.1	3579.2±1974.0	<.001
LPA (min/day)	56.4±16.2	49.0±14.6	30.5±15.0	<.001
MPA (min/day)	21.0±15.5	17.7±9.2	7.1±6.2	.001
VPA (min/day)	1.3±1.6	1.2±1.4	0.5±0.6	.186

LPA: low intensity physical activity; MPA: moderate intensity physical activity; VPA: vigorous intensity physical activity

### Odds ratio of osteopenia according to the ASM index and physical activity

Table 4 showed the calculated result of ORs to osteopenia and osteoporosis in the normal and osteopenia group based on the ASM index. The osteopenia group (OR=4.823, *P*=.042) was significantly higher based on the normal group. Also, the OR calculated after classifying into the active and inactive group, based on the physical activity recommendations (150 minutes per week) presented by WHO showed significantly higher level in the inactive group even after adjusting age (OR=5.478, *P*=.005 and OR=4.816, *P*=.013 respectively).

**Table 4.** Odds ratio of femur BMD status to ASM index, BMI and physical activity

Variables	OR (95% CI)	*P*	OR^a^ (95% CI)	*P* for linear trends
ASM index				
Normal	1		1	
Sarcopenia	4.823 (1.059-21.965)	.042	4.420 (.962-20.315)	.056
Physical activity				
Active	1		1	
Inactive	5.478(1.666-18.014)	.005	4.504 (1.333-15.217)	.015

OR^a^ was adjusted for age

OR: odds ratio; CI: confidence interval; BMD: bone mineral density; ASM: appendicular skeletal muscle mass; BMI: body mass index

## DISCUSSION

The main purpose of this study was to verify the correlation between femoral BMD and lean mass that is a sub-concept of body composition and body fat and determine the role of physical activities on the BMD through the objective investigation in 119 elderly women. The classification results of the groups on the basis of the femur BMD of the subjects showed that the ratio was 29.4% (n=35) in the normal group, 58.8% (n=70) in the osteopenia group, and 11.7% (n=14) in the osteoporosis group. This value was lower than the value shown in the domestic and foreign large-scale epidemiological survey and previous studies2. These differences in prevalence resulted from the subject selection in this study conducted in the elderly people who had no specific medical diseases and were able to perform a normal daily lifestyle.

Recently, increases in elderly population has led to an increased interest in age-related diseases. Among these diseases, musculoskeletal disorders are well recognized as a serious problem. The loss of BMD in old age leads to an increase in fractures, disabilities, hospitalization and mortality with increases in risk of the secondary damage. In particular, women are known to be more vulnerable to osteoporosis due to the loss of the BMD^20^. Several studies have reported that factors associated with body composition play an important role in maintaining BMD in old age, but which component of the lean mass and body fat plays a role on the BMD remain controversial^21,22^. The comparison result of the body composition and obesity indicators in accordance with the BMD and groups in this study showed that there was a significant linear trend in body weight, BMI, lean mass, and ASM index, but there was no significant difference in waist circumference and percent of body fat among groups. Lim et al. in the study on the correlation between the calcaneus BMD and body composition in 402 domestic adults aged over 45 years old, reported that body weight, BMI, lean mass had a significantly positive correlation with BMD in both men and women^23^. These results are similar to the study conducted by Walsh et al. on the association between the systemic BMD and sarcopenia in 213 US adult women who reported that the ASM index had a significantly positive correlation similar to the systemic BMD in menopausal women^24^. In addition, the obesity index was interpreted in the context that that lean mass had a significant correlation with the BMD, but body fat had no significant association with the BMD, as reported by Douchi et al.^25^. In this study, it was also verified that not body fat, but lean mass among body composition had a significant correlation with osteopenia. This is because muscle plays a positive role in bone metabolism through myokine such as IGF-1 (insulin-like growth factor-1) and FGF-2 (basic fibroblast growth factor-2) etc., but this also resulted from the complex roles of positive function of adipokine in fat and a concurrent negative function of the inflammatory cytokine^26,27^. However, an increase in lean mass plays a positive role in the prevention of osteoporosis, but body fat plays a native role^28^. On the other hand, another study showed that an increase in both lean mass and body fat plays a positive role in the prevention of osteoporosis^21^. Thus, the role of the lean mass and body fat requires verification through a more quantifiable method and the metabolic pathway of the bone metabolism.

On the other hand, physical activities in old age not only reduce death rate caused by chronic diseases regardless of its form but also can induce the maintenance and increase of the BMD through the physical load and bone metabolism–related biochemical markers^15^. A large-scale epidemiological and cross-sectional study reported a positive correlation between physical activities and BMD. However, quantitative surveys on the physical activities in old age are insufficient because physical activity survey is limited to only questionnaire in the most of the previous studies. In this study, the results that physical activities among groups according to the BMD was investigated through the accelerometer showed that there is a significant linear trend among groups in the daily of step, low intensity physical activities, and moderate intensity physical activities. In the comparison of the physical activity degree by each group, the number of the daily average steps was 7,411 in the normal group, 6,500 in the osteopenia group, and 3,580 in the osteoporosis group and the low-intensity physical activities was 56 minutes in the normal group, 49 minutes in the osteopenia, and 30 minutes in the osteoporosis group. In addition, moderate intensity physical activities was 21 minutes in the normal group, 18 minutes in the osteopenia, and 7 minutes in the osteoporosis with no significant difference among groups in high intensity physical activities. These results were similar to the results of Chastin et al. and Gaba et al. of the positive role of the reduction in the sedentary time, regular exercise and physical activities play in maintaining the BMD^29,30^. Thus, an increase in physical activities and a performance of the regular walking exercise can be an effective method for preventing the BMD.

In addition, the calculation results of the OR about the osteopenia and osteoporosis after dividing into the sarcopenia group and normal group based on the sarcopenia cutoff value of previous domestic studies showed that a probability exposed to osteopenia and osteoporosis was higher by about 4.8 times in the sarcopenia group than the normal group^4^. The calculation results of the OR of the osteopenia and osteoporosis after classifying into the inactive group and active group based on the weekly elderly physical activities by WHO showed that a probability exposed to osteopenia and osteoporosis was 5.4 fold higher in the group with less than 150 minutes of weekly physical activity, as compared to the group with more than 150 minutes of the weekly physical activity^19^. This result showed that regular physical activity is effective for the maintenance of muscle mass and muscular strength and induces the synthesis of vitamin D through outdoor activities, suggestive to play a positive role in maintaining BMD^31,32^.

## CONCLUSION

Overall, preservation of muscle mass plays as a preventive means against osteopenia and/or osteoporosis in elderly women. In addition, physical activity, especially strength training, plays a positive role in maintaining appropriate bone mass in old age. Therefore, the current findings of the study suggest that the maintenance of muscle mass via physical activity and/or a healthy nutrition should be promoted as a preventive strategy against osteopenia and osteoporosis in elderly women. Finally, we recognize limitations of the study not considering dietary intakes and outdoor activities; hence, it is necessary to conduct further research on dietary surveys and outdoor activities in the future.
